# Promoting patient engagement with self-management support information: a qualitative meta-synthesis of processes influencing uptake

**DOI:** 10.1186/1748-5908-3-44

**Published:** 2008-10-13

**Authors:** Joanne Protheroe, Anne Rogers, Anne P Kennedy, Wendy Macdonald, Victoria Lee

**Affiliations:** 1 National Primary Care Research and Development Centre, Fifth Floor Williamson Building, University of Manchester, Oxford Road, Manchester, UK

## Abstract

**Background:**

Patient information has been viewed as a key component of self-management. However, little attention has been given to methods of dissemination or implementation of effective information strategies. Previous problems identified with the use and implementation of patient information point to the need to explore the way in which patients engage with and use information to support self-management for chronic conditions.

**Methods:**

Four published qualitative studies from a programme of research about self-management were analysed as a group; these included studies of the management of inflammatory bowel disease (IBD); self-help in anxiety and depression (SHADE); menorrhagia, treatment, information, and preference (MENTIP) study; and self-help for irritable bowel syndrome (IBS). For the analysis, we used an adapted meta-ethnographic approach to the synthesis of qualitative data in order to develop an evidence base.

**Results:**

The ontological status and experience of the condition in everyday life was the most dominant theme to emerge from this synthesis. This, coupled with access to and experience of traditional health services responses, shaped the engagement with and use of information to support self-management. Five key elements were found which were likely to influence this: the perception and awareness of alternative self-management possibilities; the prior extent and nature of engagement with information; the extent of and ability to self-manage; opportunities for use of the information and the stage of the illness career; and congruence and synergy with the professional role.

**Conclusion:**

People with chronic conditions need support from providers in both supply and engagement with information, in a way which gives legitimacy to the person's own self-management strategies and possible alternatives. Thus, a link could usefully be made between information offered, as well as patients' past experiences of self-management and engagement with services for their condition. The timeliness of the information should be considered, both in terms of the illness career and the type of condition (*i.e*., before depression gets too bad or time to reflect on existing knowledge about a condition and how it is to be managed) and in terms of the pre-existing relationship with services (*i.e*., options explored and tried).

More considered use of information (how it is provided, by whom, and at what point it should be introduced) is key to facilitating patients' engagement with and therefore use of information to support self-management.

## Background

Patient information has been viewed as a key component of self-management, and recent official policy identifies health information as important for patients 'so that they are better able to care for themselves and their families' [1]. This sentiment is encompassed in an initiative designed to provide information prescriptions [2], whereby information designed to support and signpost patients to relevant and personal sources of information about services and treatments required at key points in the 'care journey' (*e.g*., diagnosis, stages of treatment, care planning, and discharge from hospital) is provided by health professionals to patients and caregivers,

The enthusiasm for proliferating the use and deployment of information in health systems is supported by studies and reviews of patient information, which suggest the existence of considerable demand for better quality information and evidence that written information increases knowledge. However, evidence that information on its own promotes informed choice or leads to changes in behaviour is poor [3], and little attention has been given to methods of dissemination or the implementation of effective information strategies. There has been recognition in official health policy of the need to address barriers that are viewed as inhibiting the access to and use of information, for example, through initiatives aimed at improving health literacy. Low levels of literacy and numeracy are viewed as having an adverse impact on health and well-being and contributing to a growing health gap between those who are 'empowered' to help themselves and those that are not. The current focus of action to reduce the health literacy gap is predicated on making information easily accessible and comprehensible.

Policies about patient information are also linked to the notion of empowerment, and a common goal in policy statements about self-management is for patients to become active agents taking charge of their own health and their interactions with health services [4]. Empowerment in this context is used in a general sense to focus on individual actions in engaging with health care and in health care settings, and is closely aligned to a social psychological perspective of individuals' developing control over their own lives [5] (*e.g*., as in patient activation). The latter implies that empowerment should be viewed principally as an outcome of patient information policies, leaving the process of how this may be achieved under-elaborated. Moreover, terms such as 'empowerment' and self-efficacy have assumed an increasingly normative tone when used in the context of supported self-management initiatives. It has come to mean what patients 'should' do rather than a neutral observation of what patients might actually do to engage with information in a given set of circumstances [6]. A more open and less judgemental perspective toward those with a chronic condition has been suggested [7].

Notions of health literacy alone are unlikely to address the full range of influences impacting on information uptake and utilisation by individuals. Regardless of literacy levels, peoples' interpretations of information are seemingly influenced by prior knowledge, expectations values, and preferences [8]. Dixon-Woods points to a mechanistic model of communication and a predominance of biomedical concerns in patient information, and portrays patients as passive and open to manipulation [9]. Where patients' views are directly sought about information, their concerns reflect a patient empowerment rather than patient education discourse indicating the importance of involving patients in the design and content of information from the outset [10]. The degree of complexity of living with a chronic condition, and the failure to ground this in the lived experience of patients, has been identified as a notable weakness in patient information leaflets designed to aid patient self-management [11]. There is a paradox in the current 'health information age' where on the face of things patients have greater access to health information than ever before (*e.g*., world wide web, patient internet sites, access to on-line medical journals, etc.), but large social disparities exist in levels of access, use, and understanding of this information. One of the reasons why some patients do not engage with or use such opportunities is related to a lack of perceived utility and pertinence of such information for managing health and health care [12]. How and who provides information, its part in the dynamic nature of the relationship with the health care professionals (HCPs), and the point at which it is introduced are likely to be salient factors in uptake [13].

Problems identified with the use of patient information point to the need to further explore the way in which patients engage with and use self-management information. In particular there is a lack of understanding of the way in which people use information in health contexts relating to the self-management of specific long-term conditions. Drawing on a set of four qualitative studies from a programme of research about self-management, the analysis here focuses on the uptake of information for long-term conditions in order to address our new research question, 'What influences patient engagement with information to support self-management for chronic conditions?'

## Methods

Our research has focused on exploring the contexts and influences under which patients and clinicians are likely to engage in a shared approach to self-management within a health service organisational environment. We have used an adapted meta-ethnographic approach to the synthesis of qualitative data in order to develop an evidence base. In a series of qualitative studies of aspects of self-management, the use of information and how patients engage with information to support self-management were explored as elements of self-management support interventions that were subject to evaluation [14].

Meta-synthesis is a technique for the systematic interpretation and reinterpretation of qualitative studies. It allows new insights and understandings to emerge through a process of a re-conceptualisation of themes from secondary qualitative analysis of existing qualitative data sets and reviews of published qualitative papers. The meta-ethnographic synthesis of qualitative data involves the systematic identification of relevant studies, data extraction, appraisal, and synthesis [15]. The inclusion criteria used for our data were all the studies conducted within our research programme on self-management that related to patient information in the primary care context. In the analysis presented here we draw on four qualitative data sets as our data source: Rogers and Kennedy, Inflammatory Bowel Disease (IBD) study [16]; Mcdonald *et al*., Self-help in Anxiety and Depression (SHADE) study [17]; Protheroe *et al*., Menorrhagia, Treatment, Information and Preference (MENTIP) study [18,19], and Rogers *et al*., Irritable Bowel Syndrome (IBS) study [20] (details of the studies are summarised in Table 1, and in the results section). Taking the qualitative studies already published together with a secondary analysis of qualitative data we drew from original transcripts and using a meta-ethnographic approach we discussed and analysed these as a group.

**Table 1:** Studies included in analysis

Source	Aims	Context and data collection	Sampling and participants	Main findings
Paper 1: Rogers and Kennedy, [16]	Qualitative study within an RCT, assessing a self-help guidebook and patient-centred consultations in IBD. Patients were given an information guidebook by their hospital consultant, and a written self-management plan was negotiated during the consultation.	Hospitals in the North West of England In depth interviews were conducted with 28 patients and 11 physicians	A purposeful maximum variation sample of patients	Organisation and physician factors inhibiting effective patient-centered consultations were identified. Attending to these barriers might maximize opportunities for self-management based on a therapeutic alliance with health care professionals.

Paper 2: Mcdonald *et al*., (SHADE) [17]	Qualitative study to assess the clinical and cost effectiveness of facilitated self-help vs waiting list control in the management of anxiety and depression in primary care. Patients on a waiting list for conventional psychological therapy were randomised to receive self-help material facilitated by assistant psychologists or to waiting list control.	Three psychological therapy services in Greater Manchester, United Kingdom Semi-structured interviews were conducted with 24. Further sample of 6 re-interviewed	Purposeful sample of patients who had completed the guided self-help. Further sample re-interviewed after they had been invited to attend for traditional therapy	There were important gaps between patients' expectancies of psychological therapy and their experience of the guided self-help. The effective implementation of 'minimal interventions' requires an understanding of the expectancies of patients concerning psychological therapy, in order to provide a basis for effective communication and negotiation between professionals and patients.

Paper 3: Protheroe *et al*., (MENTIP) [18,19]	Qualitative study within an RCT evaluating whether the addition of a computerised decision aid to written information improves decision-making in women consulting their GP with menorrhagia compared with written information alone.	General Practices in the North of England Semi-structured interviews were conducted with 18 patients.	The intervention group was purposefully sampled	Decisional conflict was significantly reduced using decision aid. Use of a decision aid was reported as significantly empowering to women.

Paper 4: Rogers *et al*., IBS Study, [20]	Qualitative study within a 3 armed RCT of a self-help information book in the management of IBS in primary care. Patients were randomised to receive the self-help guidebook; to receive the guidebook plus attendance at a self-help group meeting or to receive treatment as usual	Three health authorities in the North West of England Ten focus groups (total 59 patients). In depth interviews with 12 patients 1 year post intervention.	Ten facilitated self-help group meetings – focus groups The intervention groups were purposefully sampled after one year of follow-up.	IBS was transposed from a condition unsatisfactorily managed by medicine to one successfully managed within the life worlds of individuals. The design and evaluation of complex interventions should view participation as part of a process of continuity as well as change. The benefits of understanding the prior experience of managing illness and contact with health services include the acceptability and workability of complex interventions in patients' everyday lives.

Being in the position of having four research studies which all had data to contribute to our research question, 'What influences patient engagement with information to support self-management for chronic conditions?', we wanted to undertake a synthesis of this work with the purpose of achieving a greater understanding and developing a conceptual framework for the use of information in self-management, Campbell *et al*. [21] described this as: 'synthesis of qualitative research can be envisaged as the bringing together of findings on a chosen theme, the results of which should, in conceptual terms, be greater than the sum of parts.'

Our analysis was comprised of a number of steps. Unlike Campbell *et al*. [21], we had the added benefit of access to all the raw data (including transcriptions, reflective notes, and author insight about the context of the studies), so the first step of our analysis involved a secondary analysis. The raw data and published studies were re-interrogated by the individual authors in accordance with our new research question. As a group we had extensive discussions about the context of the studies and the data collection processes, and discussed quotes and transcripts. This process both identified gaps in the data and resulted in the emergence of several key concepts relating to the research question.

The next step of our analysis was informed by a meta-ethnographic approach similar to that described by Noblit and Hare [22], and used by Campbell *et al*. [21]. Whereas meta-ethnography requires key concepts to be noted down from published papers, by using the secondary analysis approach described above to develop our key concepts, we were able to return to the full data sets, and therefore none of the 'meaning in context' was lost.

The key concepts from the secondary analysis became the raw data for the synthesis. Once these concepts were defined for each individual study, they were examined in relation to the Rogers and Kennedy IBD study [16] (chosen as the point of comparison because it was the earliest study undertaken), and then across the other three studies. The aim of this was to translate the findings from one study to another in a thematic analysis to identify themes and patterns that existed in the qualitative data [22]. The final step in the analysis was to synthesize the translations in a 'line of argument synthesis' with the aim of moving from individual key concepts to an overall interpretation, which should represent a further level of conceptual development.

This combined approach had the advantages of both including the perspectives of the researchers who had originally collected the data as well as critical perspectives and distance of independent researchers [23]. Prolonged discussions bridged the gap between the researchers who had collected the primary data and the other researchers involved in the secondary analysis discussions. The former process provided details and nuances about the contexts of each of the studies and the original data collections and analysis [23]. Rigor was added by the use of the researchers not directly involved in the conduct of the original research providing a more objective account of emerging concepts and themes. Critical discussion continued until consensus was achieved. This collective data analysis process was facilitated by the authors sharing a similar conceptual framework about supported self-management research [14,24]. Analysis moved from the particular data sets of each of the four studies to a more general orientation, which compared key themes and constructs across datasets ('line of argument synthesis').

Key questions and themes were formulated, and continually tested against the narratives about information from the original transcripts, and were subject to re-formulation as part of a constant cyclical process. Thus all the authors have been involved in discussions, refinements, and final analysis [23].

## Results

The four studies considered in this synthesis were looking at aspects of supported self-management in a variety of medical conditions, which were: IBD, anxiety and depression, menorrhagia (heavy menstrual bleeding), and IBS (see Table 1).

The first step of the analysis, the secondary analysis, identified four key concepts relating to information: type of prior information seeking, timing of the provision of information, role of the professional in introducing and engaging patients with information, and engagement with and use of self-help materials.

As described in the methods above, the analysis moved from the particular data sets of each of the four studies to a more general orientation, which compared key themes and constructs across datasets. This phase is presented below and the concepts, with their origins in the papers, are described in detail, ending with a description of the 'line of argument synthesis' [21].

### Translating the key concepts across the studies (See Table 2)

**Table 2:** Translation of key concepts through the studies

	Paper 1 IBD	Paper 2 SHADE	Paper 3 MENTIP	Paper 4 IBS
Prior information seeking	Yes – aligned to consultant	Minimal	Minimal	Yes -orientated to alternatives to medical perspective

Timing of information provision important to self-management	Yes – early in illness career	Yes – before depression too severe	No, but early in illness career might impact on quality of life	No, but need for episodic 'just in time' information

Role of professional important	Yes – to clarify and confirm existing relationship and decisions	Yes – as gateway to use of the information and legitimisation of condition and strategies	No, but gave medical permission to use the information	No, but ensured legitimisation and permission to use information to self-manage

Engagement with and use of self-help materials	Yes – identified with others' experiences. Used to monitor, make management changes and raise awareness of condition with others. Also to fill gaps in knowledge.	Yes – identified with collective experiences of others, but severity impaired engagement. Used to monitor, make management changes and raise awareness of condition with others. 'Pick and mix' use and support whilst waiting for traditional therapy.	Yes, but linked to legitimisation of condition -led to enhanced control and ownership of knowledge. Used to support future decision-making and fill gaps in knowledge.	Yes – identified with collective experiences of others. 'Pick and mix' use. Refresher for self care action.

#### Type of prior information seeking

##### Paper one

Rogers and Kennedy IBD study [16]. This study sought to illuminate the findings of a randomised controlled trial assessing the impact of a package comprising a self-help guidebook and patient-centered consultations on disease management and satisfaction in IBD. Patients were given an information guidebook by their hospital consultant, and a written self-management plan of action was negotiated during the consultation. In-depth interviews were conducted with twenty-eight purposefully selected patients and eleven physicians.

In relation to prior information seeking, when participants came into the study they felt that they had already had most of the information they needed from their consultant. Thus, information seeking from these individuals was closely aligned to information previously received from consultants.

##### Paper two

Mcdonald *et al*., SHADE study [17]. This study aimed to assess the clinical and cost effectiveness of facilitated self-help versus waiting list control in the management of anxiety and depression in primary care. Patients on a waiting list for conventional psychological therapy were randomised to receive self-help material facilitated by assistant psychologists or to waiting list control. Twenty-four semi-structured interviews were conducted with patients who had completed the guided self-help. In addition, six of the twenty-four were re-interviewed after they had been invited to attend for traditional therapy.

Information seeking did not seem to be a central theme for participants in this study. Only a minority of participants reported actively searching for information, and information seeking overall seemed to be inhibited due to the lack of formulation of depression as a specific condition in itself.

##### Paper three

Protheroe *et al*., MENTIP study [18,19]. This study aimed to explore the findings of a randomised controlled trial comparing an interactive, computerised decision aid to written information in women consulting their general practitioner with heavy periods (menorrhagia). Eighteen patients in the intervention group were purposively sampled and interviewed.

Within the participants in this trial, there was minimal prior information seeking as the condition was not perceived as one for which treatment options were available. In other words, information was not actively sought, as there was a general feeling of accepting that 'that's my lot'.

'. . they usually say, "Well its just women's things isn't it?"' [ID C13012, MENTIP study]

##### Paper four

Rogers *et al*., IBS Study [20]. This study was a nested qualitative study within a three-armed randomised controlled trial of a self-help information book in the management of IBS in primary care. Patients were randomised to receive the self-help guidebook; to receive the guidebook plus attendance at a self-help group meeting, or to receive treatment as usual. Qualitative data was taken from two sources: ten facilitated self-help group meetings (with a total of fifty-nine participants), and twelve interviews with patients from the intervention groups that took place after one year of follow-up.

There was a general feeling of failure of traditional services to meet needs from the participants in this study with respect to medical input, and thus there was a high level of active critical information seeking orientated to finding alternatives to the traditional medical perspective and ways to self-care.

'I was given a sheet when I was first diagnosed in hospital, so. . . , and it was literally like, don't eat anything. . . (laughter) just drink water for the rest of your life.' [Group1, IBS study]

#### Timing of the provision of information

For both the IBD [20] and the SHADE [17] studies, it was found that for maximum utility the timing of the provision of information should be relatively early on in the illness career:

'Well to me it really wasn't that useful because by then I had had this problem for seven years and seeing different doctors and doing my own study and whatever, so by then I knew what's what anyway. I mean it's a great book when you go on the first visit – its excellent for that first visit – but for someone who's been ill for eight years – no. . . I wish I had been given that book when I went to see them the first time.' [ID28, male, IBD study]

In the case of the IBD study [20] the information was given to the patient by the HCP. The information was then linked to the HCP and had the effect of reinforcing a pre-existing understanding and relationship between the HCP and the patient. Additionally, the information format indicated areas where patients' choice might influence treatment decisions and possibly led to improved outcomes because a written self-management plan supported earlier self-treatment of exacerbations.

With the SHADE study [17], there were suggestions that the provision of the self-help information was likely to have most impact early in the illness trajectory. Patients felt there was a point in the illness trajectory beyond which they would struggle to engage with or use information if their symptoms were too severe or too chronic:

'If I'd gone right smack-bang in the middle of my depression. . . when it was bad, I'd have felt cheated on four sessions.' [ID 124, SHADE study]

Conversely, due to the nature of the medical conditions in the studies, timing of information provision did not play such an important role in either the MENTIP study [18,19] or the IBS study [20]. In the study by Protheroe *et al*. [18,19], the episodic nature of menorrhagia led to the finding that the timing of information (in the form of the interactive decision aid) was not felt to be critical in terms of physical health, as this condition does not deteriorate over time. However, although there would be a benefit to receiving such information at any point in the illness trajectory, earlier decisions made as a result of the information could have a benefit in terms of earlier improvements in quality of life. Similarly in the study by Rogers *et al*. [20], due to the nature of IBS, which also does not deteriorate with time, the timing of provision of information, with regard to the illness trajectory, was not felt to be critical. Rather, in this study patients described the episodic need for this information in times of symptom exacerbations. There was a requirement for this information to be available 'just in time':

'. . . when it does flare-up, I get the book out and I read it and it gets things into perspective again, because you do get things out of perspective I think, when it's bad.' [Group 2, IBS study]

**Figure 1:**
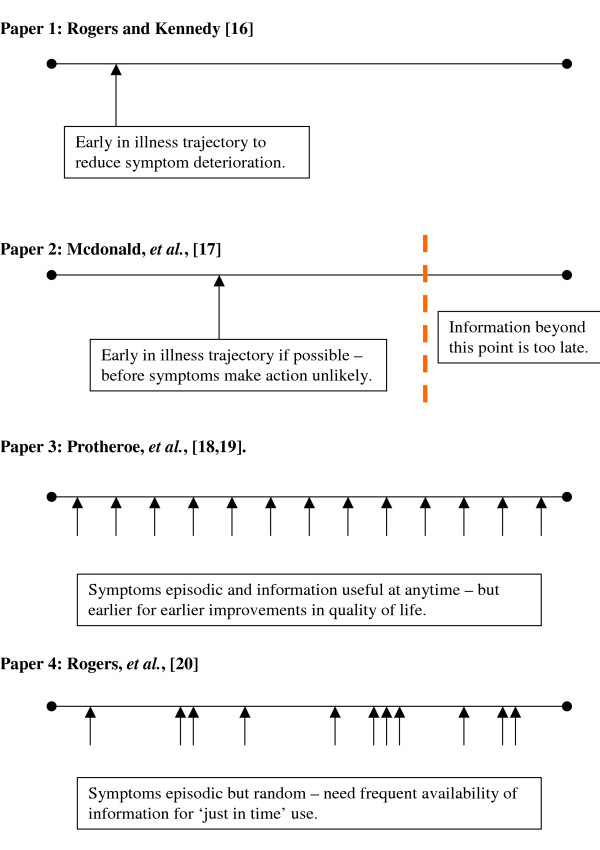
Timing of information/illness trajectories.

Looking across the four studies, Figure 1 represents how the timing of information provision is linked to the context of the nature of the medical condition.

### Role of the professional in introducing and engaging people with information

In the IBD study [20], the role of the health professional, in the form of the patient's hospital consultant, was central to giving out the guidebook and the provision of primary legitimization of self-management. Use of the guidebook was incorporated into the consultation as part of the study intervention, and joint reference to the guidebook was used to re-emphasise, clarify, and confirm information relating to self-management in the context of the existing relationship. Similarly, in the SHADE study [17], a health professional, the assistant health psychologist, was the gateway into making the self-management information both accessible and a source of emotional and self-help support.

'. . if you were feeling particularly down, you would chuck that manual at the back of the drawer and just left it there. The combination of the two (manual and facilitation) is very, very good. One thing you could chat through how you felt with somebody who had a particular level of professional understanding and also you have the manual there. There's no point in having one without the other I don't think.' [ID 001, SHADE study]

The involvement of the health professional also provided legitimization of the condition and the strategies outlined in the information guidebook.

Conversely, in the MENTIP study [18,19], there was no direct role for the health professional. Rather, permission was needed from a patient's general practitioner (GP) before being recruited into the study, and this permission gave the decision-making information package 'medical respectability' from a recognized source. In the IBS study [20] the role of the health professional was similar to the MENTIP study, as trial participants were accessed through their GP thus ensuring permission and legitimation of the information source. The information was used by patients as a way to help them move from dependence on medical management of their condition to using an informational, self-help approach.

### Nature of engagement with and use of self-help materials

The nature of the medical condition was again found to be important in how patients engaged with and used information. In the IBD study [20] the condition is a diagnosed 'mainstream' medical condition. Patients engaged with the self-help materials as they identified with the experiences of others described within the materials, which also had the function for some patients of 'normalising' the condition. The materials were also seen as confirmation of the seriousness of the condition. In the SHADE study [17], depression was a diagnosed condition and carries a stigma. The condition of IBS is diagnosed by a process of exclusion of all other possible causes of the symptoms suffered and is experienced by the patients as medically unexplained [20]. However, both these latter two studies also engaged with the self-help materials as sources of collectivisation of experiences and identification with other patient stories:

'. . if I were doing anything or I fancied eating anything, then I went through the book to see what anybody else had said about it, you know, and I thought, 'they've tried it and it's done so-and-so, I'll try it and see what happens with me' and then if it works the same way as what the book said, I thought, 'right, that's it', I put a little tick at the side of it as if to say you don't try that again.' [Group 1, IBS study]

This was less noticeable in the MENTIP study [18,19]. Some participants (and indeed their health professionals) had considered their condition as part of the normal range of menstrual bleeding, and something with which they just had to contend. In the case of the MENTIP study, engagement with the information was related to the 'medical respectability' of the information itself. However, the fact that heavy menstrual bleeding was given a medical name (menorrhagia), and that treatments were discussed, conferred some legitimacy to the condition, which in turn improved engagement with the information. The severity of the medical condition was also seen as an impediment to using self-help materials. In the SHADE study, this difficulty was exacerbated by the low levels of energy and impaired ability to concentrate that accompanied depression.

The guides were used in different ways by patients. In the IBD study [20] and the SHADE study [17], the information guides were used with family and friends to raise awareness of the condition and individually to monitor their own condition and make management changes as necessary. In both the IBD study [20] and MENTIP study [18,19], the information was used to fill in perceived gaps left by medical knowledge or to provide support not provided by the traditional approach to treatment from services. In the MENTIP study this information gave women a feeling of control and ownership of information that was used to support current or future treatment decision making; whereas in the IBD study the information was used more as a guidebook and diary to understand the antecedents of exacerbations.

'I was umming and ahhing whether to do anything or whether not to do anything, and what would be the best option for me. Now if my periods do go worse again then I will go back to the GP and ask to go on the coil. . . I would say its reduced uncertainty cos I know there's something there if I need to do something.' [ID 112011, MENTIP study]

In the IBS [20] and SHADE [17] studies, the guides were used in an eclectic fashion as a refresher for reference for self-action at times of relapse or for maintenance of their condition. In the SHADE study [17], they were used as support while waiting for traditional therapy, and the depth to which the guide was used varied; democratic use of information allowed readers to skim or go to great depth (*e.g*., cognitive restructuring).

### Line of argument synthesis

The line of argument synthesis, according to Noblit and Hare [22], entails the construction of an interpretation which 'serves to reveal what is hidden in individual studies and to discover a whole among a set of parts.'

**Figure 2:**
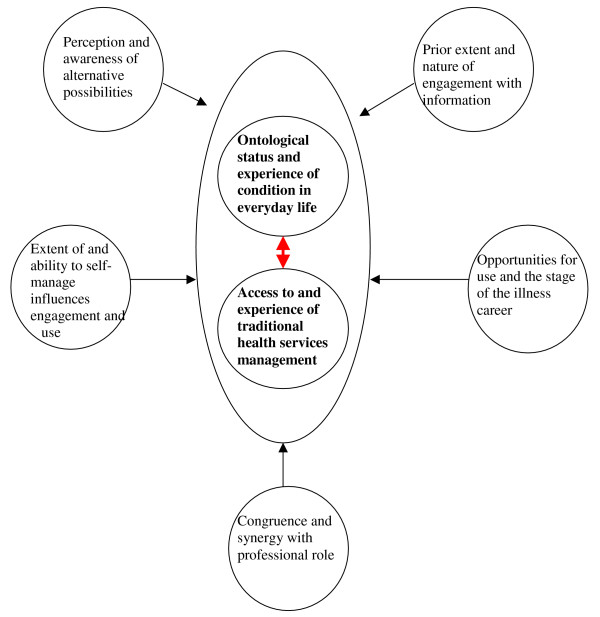
Line of argument synthesis: the key influences that determine patient engagement with self-management information.

Examining the themes and patterns emerging across all the datasets, what emerged from the synthesis were the ontological status and the experience of the condition in everyday life. This, coupled with access to and experience of traditional health services responses, shaped the engagement with and use of information to support self-management. The 'ontological status' of the condition refers to the nature and status (legitimacy) of the condition in terms of how individuals experienced it, how socially acceptable it was considered to be, the previous way it had been managed by themselves, and the impact on peoples' everyday lives. The synthesis indicated that establishing a relationship about the nature of the condition and the way it ought to be managed between patient and professional is relevant to establishing active engagement with information and its perceived subsequent utility. Professional engagement with patients about self-management information seemingly gave patients 'permission' to engage with and use information that they felt had been sanctioned by health care. These two themes were relevant to other elements associated with engagement with and the way in which the information was used (see Figure 2).

#### Perception and awareness of alternative possibilities

• Self-management information may be more likely to influence awareness of alternative possibilities in medically unexplained conditions such as IBS or menorrhagia where people have experienced little help from their HCP because there are, or they believe there to be, no medical treatments available.

#### Prior extent and nature of engagement with information

• People with certain conditions seem less likely to search for and engage with information, for example, those with menorrhagia and depression. Where there is a view that a condition has no treatment or has a certain stigma attached to it, then people are less likely to think about searching for self-management information.

#### Extent of and ability to self-manage influences engagement and use

• Some conditions are more amenable to patient-initiated use of self-management information than others. For example, in IBS self-management is generally the only option. For IBD and depression opportunities for self-management need to be 'brokered' by a health professional.

#### Opportunities for use and the stage in a particular illness career

• Certain conditions may respond better to early use of self-management information – for example, IBD and depression, where there is a likelihood that the condition will worsen over time if nothing is done. For episodic conditions, self-management information is there when needed.

#### Congruence and synergy with professional role

• Professional permission to use self-management information can encourage uptake by those with stigmatized or uncertain conditions such as IBS or menorrhagia. For conditions where medical treatment dominates such as IBD, uptake and use of self-management information is best achieved through a partnership approach.

As was described in the methods section, the purpose of a synthesis of qualitative datasets was to move from the level of individual key concepts to an overall interpretation, which should represent a further level of conceptual development. We believe the framework proposed in Figure 2 represents this further level of conceptual development and answers our research question, 'What influences patient engagement with information to support self-management for chronic conditions?' The framework suggests that there are several key factors involved that are likely to promote the engagement and implementation of a strategy for promoting engagement with supported self-management information.

## Discussion

In this paper, we have used innovative methodology to synthesize four individual qualitative studies in the area of self-management. We have re-interrogated the data with a new research question, and then used a meta-ethnographical approach to synthesize the findings across the four studies. Our synthesis has provided novel insights into engagement with information. First, we highlighted four key concepts relating to information that are common to people with various medical conditions. However, by using a line of argument synthesis, we additionally found that people with different medical conditions appear to respond to self-management information in different ways, and that this was affected by their access to and previous experience of traditional health services. These new insights into engagement with information were not apparent in any of our individual studies and would not have come to light in a narrative review.

A particular strength of our approach was the involvement of all the authors of the individual studies, giving us access to all the raw data, reflective notes, and the perspectives of the individuals who originally collected the data. Often, reviews of this type of data are restricted by necessary shorter publications, leading to possibly less than adequate descriptions of methods and results. This strength could also be construed as a limitation, *i.e*., how can we judge the quality and rigor of our synthesis? As was described in the methods, we attempted to achieve rigor by the use of the researchers not involved in the conduct of the original research, providing a more objective account of emerging concepts and themes. Critical discussion was continued until consensus was achieved; however, a further possibility would be to check the cogency and plausibility of the synthesis with researchers who are distant from the original papers.

A further limitation to this research is that all the long-term conditions studied, with the possible exception of IBD, are not generally associated with life-threatening illness. More research would be needed to consider engagement with information in the presence of significant physical illness, such as severe chronic obstructive pulmonary disease. However, some parallels could be drawn; it seems likely that the timing of information in relation to the particular stage in the illness career would be of increased significance with a chronic disease that is likely to deteriorate significantly.

As was stated in the methods, the purpose of this research was to identify factors which influence patient engagement with information to support self-management for chronic conditions, with the aim of achieving greater understanding in order to develop a conceptual framework for the use of information in self-management. These findings suggest that professionals wishing to support self-management need to carefully consider the nature of the chronic condition in relation to how information can be used most effectively. For example, people with conditions which could be considered medically unexplained or uncertain, such as IBS, or menorrhagia, may not be aware of information or support, and may also benefit from professional permission to encourage the use of self-management information. People will vary, according to the nature of their condition, as to whether or not they are likely to have already looked for information, and considering the timing and stage in an illness career will be very important in relation to engagement with information, for example, early in an illness likely to deteriorate, or at the time of an exacerbation in conditions such as IBS or menorrhagia which are episodic.

Considering these influences in using information to support self-management is likely to increase the engagement with and usefulness of the information, as part of a dynamic relationship with the professional established over time, rather than a standalone strategy. These findings demonstrate that effective patient information relies on a solid understanding of a patient's needs, readiness for that information, and processes of engagement.

## Conclusion

While ready access to information that is easy to understand is important, our work indicates that self-management initiatives which involve information may be more successful in engaging people if an approach is used which is tailored to the type of condition and illness stage. People with different conditions appear to respond to self-management information in different ways. It is important to acknowledge and reflect on the status of the condition, and its impact on everyday life (*e.g*., a condition which is traditionally biomedical, one which is uncertain, or one which is likely to cause stigma).

People with chronic conditions need support from providers in both supply and engagement with information, in a way which gives legitimacy to the person's own self-care strategies and possible alternatives. Thus, a link could usefully be made between information offered and patients' past experiences of self-management and engagement with services for their condition. The timeliness of the information should be considered, both in terms of the illness career (*i.e*., before depression gets too bad, or time to reflect on existing knowledge about a condition and how it is to be managed) and in terms of the pre-existing relationship with services (*i.e*., options explored and tried). Provision of information should be considered to be part of a dynamic relationship, and not a static entity to be disseminated at one point in time as a stand alone strategy.

More considered use of information (how it is provided, by whom, and at which point it should be introduced) is key to facilitating patients engagement with and therefore use of information to support self-management.

## Competing interests

The authors declare that they have no competing interests.

## Authors' contributions

All authors participated in the conception, design, coordination of the study, and the analysis of the data. JP drafted the manuscript. AR and AK contributed to the drafting of the manuscript. All authors read and approved the final manuscript.
